# Correction to: Chiron: translating nanopore raw signal directly into nucleotide sequence using deep learning

**DOI:** 10.1093/gigascience/giz049

**Published:** 2019-05-11

**Authors:** Haotian Teng, Minh Duc Cao, Michael B Hall, Tania Duarte, Sheng Wang, Lachlan J M Coin

**Affiliations:** 1 Institute for Molecular Bioscience, University of Queensland, St Lucia, Brisbane, QLD 4072, Australia; 2 Computational Bioscience Research Center (CBRC), King Abdullah University of Science and Technology (KAUST), Thuwal, 23955, Saudi Arabia

This is a correction to: *GigaScience*, Volume 7, Issue 5, 1 May 2018, giy037, https://doi.org/10.1093/gigascience/giy037

In the original version of the article “Chiron: translating nanopore raw signal directly into nucleotide sequence using deep learning” by Haotian Teng et al. [1], the GigaDB DOI for reference 37 [2] was incorrect. In the Table 2 note, the authors amended some sentences. The reference and the Table 2 note have been corrected, and the authors apologize for the error.

## References

[1] Teng H, Cao MD, Hall MB et al. Chiron: translating nanopore raw signal directly into nucleotide sequence using deep learning. GigaScience. 2018;7(5):1–9. 10.1093/gigascience/giy037 PMC5946831 29648610

[2] Teng H, Cao MD, Hall MB et al. Supporting data for “Chiron: translating nanopore raw signal directly into nucleotide sequence using deep learning.”. GigaScience Database. 2018, 10.5524/100425. PMC5946831 29648610

