# Supplementary material for: Chiron: translating nanopore raw signal directly into nucleotide sequence using deep learning
Source: Gigascience. 2018 Apr 10;7(5):giy037. doi: 10.1093/gigascience/giy037 (PMC5946831; doi:10.1093/gigascience/giy037)
Supplement: Response_to_Reviewer_Comments_Original_Submission.pdf [file giy037_response_to_reviewer_comments_original_submission.pdf]

## Author's Response To Reviewer Comments

Reviewer #1

Query:
Speed Performance:
Your "CPU rate" tests for Albacore and Chiron seem to be using a single thread. It is confusing to include these results with the "GPU rate" tests, which presumably use an entire GPU. It would be more realistic to compare an entire GPU with an entire CPU, which on most modern systems is at least four threads, often eight or more.

Response:
We tested the CPU rate on 4 threads and 8 threads and 20 threads, and full CPU utility is observed, the basecalling speed is increased
proportionally as more threads are used, so we believe the CPU resource is used efficiently under a multi threads situation. We added a 8-
core CPU rate to table 2, and updated the table legend as follows:
“Single core CPU rate is calculated by dividing the number of nucleotides basecalled by the total CPU time for the basecalling analysis.
8 core CPU rate is estimated by multiplying single core cpu rate by 8, based on observed 100% utility of CPU processors in multi-threaded mode on 8 cores. “

Query:
In the conclusion, you state that Chiron using a GPU is "faster than current data collection speed". However, at 450 bp/sec/pore (the current Nanopore sequencing rate), Chiron would only be able to keep up with about three in-strand pores. A MinION run can generate over 5 Gbp of reads, which would take over a month to basecall using your quoted GPU rate.

Response:
We have deleted this statement as it was misleading. We have included the following paragraph in the discussion to acknowledge the speed limitations of Chiron:

“Our model is substantially more computationally expensive than Albacore and somewhat more computationally expensive than BasecRAWller. This is to be expected given the extra depth in the neural network. Our model can be run in a GPU mode, which makes computation feasible on small to medium sized datasets on a modern desktop computer. “

Query:
Consensus accuracy
In addition to the error rate metrics for basecalled reads, I would like to see error rate metrics for the consensus sequences produced by each basecaller's reads. For researchers who work with assembly or other high-read-depth analyses, consensus accuracy may be more important than individual-read accuracy.
I would suggest using either Racon or Canu to measure consensus accuracy, as they are widely used tools in the Nanopore sequencing community. I realise this would only be possible for your

bacterial and viral read sets, where depth is sufficient for assembly and sequence consensus.

Response:
Thank you for this suggestion. We have calculated the consensus rate for bacterial and viral datasets, using Miniasm + Racon. We describe the approach used in the methods section:

"We assessed the quality of assemblies generated from reads produced by different base-callers. For each base-caller, a de-novo assembly is generated by the use of only Nanopore reads for the \tb{} \ec{} and Lambda Phage genome. We use Minimap2 and Miniasm to generate a draft genome, then Racon is used to polish on the draft genome for 10 rounds.

The results are presented in Table 2 and Figure 3, and summarised in the text as follows:

"In order to assess the quality of genomes assembled from reads generated by each basecaller, we used Miniasm together with Racon to generate a de-novo genome assembly for each of the bacterial and viral genomes (see Methods). The results presented in Table 2 demonstrate that Chiron assemblies for Phage lambda and E-coli samples have approximately half as many errors as those generated from Albacore (v1 or v2) reads. For M. tuberculosis, Chiron has fewer errors than Albacore v1, but slightly more than Albacore v2. The identity rate and relative length for each round of polishing with Racon are shown in Figure 3."

Query:
I am wary about including cloud-based Metrichor results in your comparison, as they aren't replicable. Is a version number possible for the
Metrichor data? Or if (as seems likely) Metrichor uses similar code to Albacore, is there an equivalent Albacore version? At the very least, it
would be useful to provide the date the reads were basecalled in Metrichor.

Response:
We give the date when the data is basecalled in the "Parameters for basecalling" section in paper: "The data is basecalled on Metrichor on Jun 3rd 2017(Lambda), May 18th 2017(E. coli), Jun 4th 2017(M. tuberculosis), and June 20th 2017(NA12878-Human)."

Query:
The abstract says, "the first deep learning model", but then the intro says, "one of the first".
These comments seem to contradict each other.
Can you be clearer?

Response:
We now revise the statement in the introduction to say:

"In this article we present Chiron, which is the first deep neural network model that can translate raw electrical signal directly to nucleotide sequence."

We also state in the same paragraph that:

“Oxford Nanopore Technologies have also developed a segmentation free base-caller, Albacore v2.0.1, which was released shortly after Chiron v0.1.”

Query:
The performance comparison section says, "...with Chiron-BS in ??". Was this an issue in my PDF or is there missing text?

Response:
Now it's displayed correctly.

Query:
The read accuracies are shown using fractions (in the table, e.g. 0.1056) and as percentages (in the discussion, e.g. 2%). Please use a consistent formulation (my preference would be percentage).

Response:
Formulation has been changed to percentage.

Query:
I was confused by this phrase in the table caption: "against three other segmentation-based Nanopore basecallers." Albacore v2 is not segmentation-based but is in the table, so I think the caption should simply read "against four other Nanopore basecallers."

Response:
The description has been corrected.

Query:
Which version of Albacore did you use for the speed performance tests? I found v1.1.2 and v2.0.1 to have similar speed performance, but it would still be clearer to explicitly state the version.

Response:
We use v1.1.2 for speed performance, the detail has been added into the description of the speed table.

#######################################################

Reviewer #2:

Query:
In particular, it seems that the performance of Chiron is very similar to other available tools, and in many cases they seem to be very similar to e.g. Albacore-1.1 that uses the event segmentation.

Response:

This is not correct. Table 1 shows that Chiron-BS is consistently better than Albacore v1.1 on bacterial and viral genomes at the read level. Moreover, following the suggestion of reviewer one, we have investigated the assembly-level accuracy (described above). We show that Chiron is superior to Albacore (v1 but also superior to v2) in generating highly accurate assemblies. We have added the following sentence in the discussion to reflect these new results:

"Bacterial and viral genome assemblies generated from Chiron basecalled reads all had less than 0.5\% error rate, whereas those generated by Albacore had up to 0.8\% error rate. This marked reduction in error rate is
essential for generating accurate SNP genotypes, a pre-requisite for many applications such as outbreak tracking. "

These results conclusively demonstrate the benefits from removing the event segmentation step in base-calling.

Query:
Moreover, design of the deep neural network underlying Chiron is much more complex than the one used in other currently available tools. In consequence, the tool is very slow and on CPU (even if parallelized) it would be very difficult to use. When using a high-end GPU card, Chiron can process ~1600bp per second. By a conservative estimate, a MinION run produces over 30000bp per
second, so one would need approx. 19 of these GPU cards to keep up with the speed of sequencing (ONT Albacore would need about 10 CPU cores to process such run on-line according to the authors' measurements, which is a much more realistic setting). Consequently, Chiron cannot be considered a practical tool.

Response:
As indicated above, we have removed the statement that indicated Chiron could be used as a real-time base-caller. However, we reject the characterization the Chiron is not a practical tool. In certain settings, obtaining the most accurate base-calls possible is extremely important. One such example is in SNP calling, e.g. accurate identification of SNPs conferring drug resistance. The fact that Chiron leads to up to a 50% reduction in base-calling error rate makes it a valuable tool.

Moreover, there are approaches to accelerating neural networks which may be used to accelerate Chiron. We have indicated this in the discussion as follows:

"Also there are several existing methods which can be used to accelerate NN-based basecallers such as Chiron. One such example is Quantization, which reformats 32-bit float weights as 8-bit integers by binning the weight into a 256 linear set. As neural networks are robust to noise this will likely have negligible impact of the performance. Weight Pruning is another method used to compress and accelerate NN, which prunes the weights whose absolute value is under a certain threshold and then retrains the NN\cite{han2015deep}."

Query:
One interesting point of the paper is that they only used a limited amount of data for training and the network seems to generalize well. It would be interesting to explore this issue. Would using significantly more data lead to a significantly better accuracy? Is the use of training data more efficient than in the case of other available tools?

Response:
We agree that the fact that the Chiron Neural Network generalises well is an interesting feature. However, exploring this issue in depth is beyond the scope of this paper. Moreover, it would be extremely difficult to compare the generalisability of Chiron and Albacore precisely because it is impossible to 're-train' Albacore on less data, as it is a proprietary basecaller.

########

We note in response to an editorial query that Chiron is now registered in SciCrunch, RRID is SCR_015950, and this information is now included in the manuscript
