# Supplementary material for: Chiron: translating nanopore raw signal directly into nucleotide sequence using deep learning
Source: Gigascience. 2018 Apr 10;7(5):giy037. doi: 10.1093/gigascience/giy037 (PMC5946831; doi:10.1093/gigascience/giy037)
Supplement: Response_to_Reviewer_Comments_Revision_1.pdf [file giy037_response_to_reviewer_comments_revision_1.pdf]

Author's Response To Reviewer Comments

We thank the reviewers for looking at our paper a second time.
