# Supplementary material for: Chiron: translating nanopore raw signal directly into nucleotide sequence using deep learning
Source: Gigascience. 2018 Apr 10;7(5):giy037. doi: 10.1093/gigascience/giy037 (PMC5946831; doi:10.1093/gigascience/giy037)
Supplement: Reviewer_1_Report_(Original_Submission) -- Ryan Wick — 19 Nov 2017 Reviewed [file giy037_reviewer_1_report_(original_submission).pdf]

Reviewer Report

Title: Chiron: Translating nanopore raw signal directly into nucleotide sequence using deep learning

Version: Original Submission **Date:** 19 Nov 2017

Reviewer name: Ryan Wick

Reviewer Comments to Author:

Thank you for Chiron! I enjoyed reading the paper and using the tool, and I was impressed by its accuracy. I think research into basecalling algorithms is very important, as they play such a major role in the usefulness of Oxford Nanopore sequencing compared to other platforms. Chiron, and its novel neural network structure, should help to push this field forward.

I was happy with the paper overall, but I had two major comments: the speed performance metrics are misleading, and there is no data on consensus accuracy. My specific comments are below.

Speed performance

Your "CPU rate" tests for Albacore and Chiron seem to be using a single thread. It is confusing to include these results with the "GPU rate" tests, which presumably use an entire GPU. It would be more realistic to compare an entire GPU with an entire CPU, which on most modern systems is at least four threads, often eight or more.

In the conclusion, you state that Chiron using a GPU is "faster than current data collection speed". However, at 450 bp/sec/pore (the current Nanopore sequencing rate), Chiron would only be able to keep up with about three in-strand pores. A MinION run can generate over 5 Gbp of reads, which would take over a month to basecall using your quoted GPU rate.

In general, I think you need to be more upfront about the speed performance difference between Chiron and Albacore. Your results show that Chiron on a GPU is comparable in speed to Albacore on a single CPU thread. However, on a computer with 8 CPU threads and a single GPU, Albacore will be 10 times faster than Chiron, and if no GPU is available it will be more than 100 times faster. Chiron is therefore only a viable alternate to Albacore for small volumes of data.

Consensus accuracy

In addition to the error rate metrics for basecalled reads, I would like to see error rate metrics for the

consensus sequences produced by each basecaller's reads. For researchers who work with assembly or other high-read-depth analyses, consensus accuracy may be more important than individual-read accuracy.

I would suggest using either Racon or Canu to measure consensus accuracy, as they are widely used tools in the Nanopore sequencing community. I realise this would only be possible for your bacterial and viral read sets, where depth is sufficient for assembly and sequence consensus.

Minor comments

The abstract says, "the first deep learning model", but then the intro says, "one of the first". These comments seem to contradict each other. Can you be clearer?

I am wary about including cloud-based Metrichor results in your comparison, as they aren't replicable. Is a version number possible for the Metrichor data? Or if (as seems likely) Metrichor uses similar code to Albacore, is there an equivalent Albacore version? At the very least, it would be useful to provide the date the reads were basecalled in Metrichor.

The performance comparison section says, "...with Chiron-BS in ??". Was this an issue in my PDF or is there missing text?

The read accuracies are shown using fractions (in the table, e.g. 0.1056) and as percentages (in the discussion, e.g. 2%). Please use a consistent formulation (my preference would be percentage).

I was confused by this phrase in the table caption: "against three other segmentation-based Nanopore basecallers." Albacore v2 is not segmentation-based but is in the table, so I think the caption should simply read "against four other Nanopore basecallers."

Which version of Albacore did you use for the speed performance tests? I found v1.1.2 and v2.0.1 to have similar speed performance, but it would still be clearer to explicitly state the version.

Level of Interest

Please indicate how interesting you found the manuscript: An article of importance in its field

Quality of Written English

Please indicate the quality of language in the manuscript: Needs some language corrections before being published

Declaration of Competing Interests

Please complete a declaration of competing interests, considering the following questions:

- Have you in the past five years received reimbursements, fees, funding, or salary from an organisation that may in any way gain or lose financially from the publication of this manuscript, either now or in the future?
- Do you hold any stocks or shares in an organisation that may in any way gain or lose financially from the publication of this manuscript, either now or in the future?
- Do you hold or are you currently applying for any patents relating to the content of the manuscript?
- Have you received reimbursements, fees, funding, or salary from an organization that holds or has applied for patents relating to the content of the manuscript?
- Do you have any other financial competing interests?
- Do you have any non-financial competing interests in relation to this paper?

If you can answer no to all of the above, write 'I declare that I have no competing interests' below. If your reply is yes to any, please give details below.

I declare that I have no competing interests.

I agree to the open peer review policy of the journal. I understand that my name will be included on my report to the authors and, if the manuscript is accepted for publication, my named report including any attachments I upload will be posted on the website along with the authors' responses. I agree for my report to be made available under an Open Access Creative Commons CC-BY license (http://creativecommons.org/licenses/by/4.0/). I understand that any comments which I do not wish to be included in my named report can be included as confidential comments to the editors, which will not be published.

I agree to the open peer review policy of the journal

To further support our reviewers, we have joined with Publons, where you can gain additional credit to further highlight your hard work (see: https://publons.com/journal/530/gigascience). On publication of this paper, your review will be automatically added to Publons, you can then choose whether or not to claim your Publons credit. I understand this statement.

Yes
