# Supplementary material for: Chiron: translating nanopore raw signal directly into nucleotide sequence using deep learning
Source: Gigascience. 2018 Apr 10;7(5):giy037. doi: 10.1093/gigascience/giy037 (PMC5946831; doi:10.1093/gigascience/giy037)
Supplement: Reviewer_2_Report_(Original_Submission) -- Tomas Vinar — 05 Dec 2017 Reviewed [file giy037_reviewer_2_report_(original_submission).pdf]

Reviewer Report

Title: Chiron: Translating nanopore raw signal directly into nucleotide sequence using deep learning

Version: Original Submission **Date:** 05 Dec 2017

Reviewer name: Tomas Vinar

Reviewer Comments to Author:

The paper proposes and implements a new base caller for ONT MinION data called Chiron. The main claim of the paper is that by working from raw signal directly, one can avoid potential errors in event detection steps.

However, this claim is not very well supported by the results. In particular, it seems that the performance of Chiron is very similar to other available tools, and in many cases they seem to be very similar to e.g. Albacore-1.1 that uses the event segmentation. So it does not seem convincingly shown that substantial increase in accuracy can gained by removing the event segmentation.

Moreover, design of the deep neural network underlying Chiron is much more complex than the one used in other currently available tools. In consequence, the tool is very slow and on CPU (even if parallelized) it would be very difficult to use. When using a high-end GPU card, Chiron can process ~1600bp per second. By a conservative estimate, a MinION run produces over 30000bp per second, so one would need approx. 19 of these GPU cards to keep up with the speed of sequencing (ONT Albacore would need about 10 CPU cores to process such run on-line according to the authors' measurements, which is a much more realistic setting). Consequently, Chiron cannot be considered a practical tool.

One interesting point of the paper is that they only used a limited amount of data for training and the network seems to generalize well. It would be interesting to explore this issue. Would using significantly more data lead to a significantly better accuracy? Is the use of training data more efficient than in the case of other available tools?

Level of Interest

Please indicate how interesting you found the manuscript: An article whose findings are important to those with closely related research interests

Quality of Written English

Please indicate the quality of language in the manuscript: Acceptable

Declaration of Competing Interests

Please complete a declaration of competing interests, considering the following questions:

- Have you in the past five years received reimbursements, fees, funding, or salary from an organisation that may in any way gain or lose financially from the publication of this manuscript, either now or in the future?
- Do you hold any stocks or shares in an organisation that may in any way gain or lose financially from the publication of this manuscript, either now or in the future?
- Do you hold or are you currently applying for any patents relating to the content of the manuscript?
- Have you received reimbursements, fees, funding, or salary from an organization that holds or has applied for patents relating to the content of the manuscript?
- Do you have any other financial competing interests?
- Do you have any non-financial competing interests in relation to this paper?

If you can answer no to all of the above, write 'I declare that I have no competing interests' below. If your reply is yes to any, please give details below.

I declare that I have no competing interests.

I agree to the open peer review policy of the journal. I understand that my name will be included on my report to the authors and, if the manuscript is accepted for publication, my named report including any attachments I upload will be posted on the website along with the authors' responses. I agree for my report to be made available under an Open Access Creative Commons CC-BY license (http://creativecommons.org/licenses/by/4.0/). I understand that any comments which I do not wish to be included in my named report can be included as confidential comments to the editors, which will not be published.

I agree to the open peer review policy of the journal

To further support our reviewers, we have joined with Publons, where you can gain additional credit to further highlight your hard work (see: https://publons.com/journal/530/gigascience). On publication of this paper, your review will be automatically added to Publons, you can then choose whether or not to claim your Publons credit. I understand this statement.

Yes
